# Oxidative Stress in the Critically Ill Patients: Pathophysiology and Potential Interventions

**DOI:** 10.1155/2018/2353128

**Published:** 2018-03-25

**Authors:** Demosthenes Makris, Peter R. Mertens, Evangelia Dounousi, Gregory Giamouzis, Saad Nseir

**Affiliations:** ^1^Critical Care Department, University of Thessaly, Larissa, Greece; ^2^Otto von Guericke University, Magdeburg, Germany; ^3^Nephrology Department, University of Ioannina, Ioannina, Greece; ^4^Emory University Hospital, Atlanta, GA, USA; ^5^CHU Lille, Critical Care Center and Lille University School of Medicine, Lille, France

Oxidative stress constitutes a mechanism of injury seen in many disease processes [1]. Increased production of reactive oxygen and nitrogen species can induce the activation of inflammatory mediators and modification of proteins, lipids, and nucleic acids, thus contributing to cellular injury and dysfunction [2]. Ιn this respect, oxidative stress can be associated with the dysfunction of major organs and systems and might be important in the outcome of critically ill patients. Notably, clinical data have shown that sepsis survivors had a greater antioxidant potential than nonsurvivors [3].

An increased oxidative burden can affect several cellular components. A potentially notable impact in critical illness is the alteration of mitochondrial function which may be pivotal in states of increased oxygen demand or hypoperfusion, that is, shock/sepsis. Indeed, there is evidence suggesting that the severity of mitochondrial dysfunction following increased oxidative stress correlates with the severity of sepsis and has been associated with adverse outcomes in septic patients [3]. The pathogenesis of mitochondrial dysfunction is rather complex. Enhanced production of reactive oxygen species may lead both to mitochondrial dysfunction and damage by the initiation of apoptotic events via the caspase pathway while nitrogen oxide metabolic products may exhibit inhibitory actions in the mitochondrial respiratory chain by interrupting ATP production reactive nitrogen leading to further mitochondrial dysfunction [4]. Thus, impaired O_2_ utilization from dysfunctional mitochondria may represent the generator of fundamental abnormalities in critical illness.

Those abnormalities at the cellular level may have significant implications in the function of organs which are frequently impaired in critical care patients such as the heart or the kidneys. Increased oxidative stress can induce ultrastructural and functional changes in cardiomyocytes that may result in altered electrical properties and compromised contractility. Intriguingly, experimental treatment with antioxidant vitamins alleviated both the systemic and myocardial inflammatory cytokine responses and decreased myocardial apoptosis [5]. On the other hand, acute or chronic kidney disease represents extraordinary states of oxidative stress which may be further exaggerated by diagnostic and therapeutic challenges which are common scenarios in critical care such as iron infusions, contrast medium application, dialysis filter exposure, artificial nutrition, and the loss of antioxidants during renal replacement therapy. Deranged kidney function directly translates into skewed adjustments in electrolyte and body water homeostasis, acid-base composition, calcium-phosphate metabolism, and erythropoiesis further resulting in accelerated atherosclerosis and disproportionally increased cardiovascular burden [6, 7].

This special issue may stimulate our effort to thoroughly understand the underlying mechanisms of oxidative stress and to reveal its impact on organ functions and systemic homeostasis. The increasing knowledge of its pathogenesis and impact on several aspects of critical illness may provide further insight in our perception of the development and management of critical illness and in the evaluation of novel concepts of treatment at the clinical level. As always, this constitutes a work in progress with exciting prospects.

*Demosthenes Makris* *Peter R. Mertens* *Evangelia Dounousi* *Gregory Giamouzis* *Saad Nseir*
